# (*Z*)-Ethyl 2-chloro-2-[2-(4-methyl­phen­yl)hydrazinyl­idene]acetate

**DOI:** 10.1107/S1600536812046521

**Published:** 2012-11-24

**Authors:** Abdullah M. Asiri, Muhammad Nadeem Arshad, Mohie E. M. Zayed, Khalid A. Alamry, Tanveer Hussain Bokhari

**Affiliations:** a Chemistry Department, Faculty of Science, King Abdulaziz University, PO Box 80203, Jeddah 21589, Saudi Arabia; b Center of Excellence for Advanced Materials Research (CEAMR), Faculty of Science, King Abdulaziz University, PO Box 80203, Jeddah 21589, Saudi Arabia; c Department of Chemistry, Government College University, Faisalabad 38000, Pakistan

## Abstract

The mol­ecule of the title compound, C_11_H_13_ClN_2_O_2_, is approximately planar (r.m.s. deviation = 0.099 Å for non-H atoms) and adopts a *Z* conformation about the C=N double bond. In the crystal, mol­ecules are linked by N—H⋯O and C—H⋯O hydrogen bonds to the same O-atom acceptor, forming zigzag chains propagating along [010]. These inter­actions give rise to *R*_2_^1^(6) loops.

## Related literature

For related structures, see: Asiri *et al.* (2011 ▶, 2012 ▶).
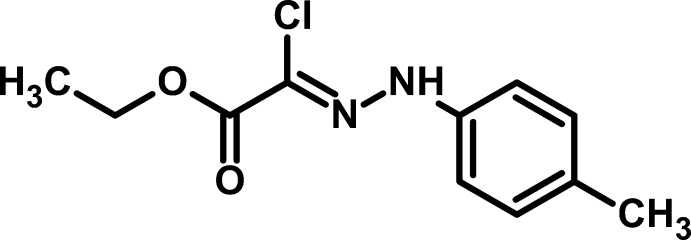


## Experimental

### 

#### Crystal data

C_11_H_13_ClN_2_O_2_
*M* *_r_* = 240.68
Monoclinic, 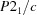

*a* = 4.6152 (1) Å
*b* = 9.9444 (1) Å
*c* = 26.3152 (3) Å
β = 90.692 (1)°
*V* = 1207.66 (3) Å^3^
*Z* = 4
Cu *K*α radiation
μ = 2.71 mm^−1^
*T* = 296 K
0.41 × 0.14 × 0.13 mm

#### Data collection

Agilent SuperNova (Dual, Cu at zero, Atlas) CCD diffractometer
Absorption correction: multi-scan (*CrysAlis PRO*; Agilent, 2012 ▶) *T*_min_ = 0.692, *T*_max_ = 1.000
9526 measured reflections
2436 independent reflections
2224 reflections with *I* > 2σ(*I*)
*R*_int_ = 0.019

#### Refinement

*R*[*F* ^2^ > 2σ(*F* ^2^)] = 0.040
*wR*(*F* ^2^) = 0.113
*S* = 1.06
2436 reflections
150 parameters
H atoms treated by a mixture of independent and constrained refinement
Δρ_max_ = 0.28 e Å^−3^
Δρ_min_ = −0.23 e Å^−3^

### 

Data collection: *CrysAlis PRO* (Agilent, 2012 ▶); cell refinement: *CrysAlis PRO*; data reduction: *CrysAlis PRO*; program(s) used to solve structure: *SHELXS97* (Sheldrick, 2008 ▶); program(s) used to refine structure: *SHELXL97* (Sheldrick, 2008 ▶); molecular graphics: *PLATON* (Spek, 2009 ▶); software used to prepare material for publication: *WinGX* (Farrugia, 2012 ▶).

## Supplementary Material

Crystal structure: contains datablock(s) I, global. DOI: 10.1107/S1600536812046521/hb6984sup1.cif

Structure factors: contains datablock(s) I. DOI: 10.1107/S1600536812046521/hb6984Isup2.hkl

Supplementary material file. DOI: 10.1107/S1600536812046521/hb6984Isup3.cml

Additional supplementary materials:  crystallographic information; 3D view; checkCIF report

## Figures and Tables

**Table 1:** Hydrogen-bond geometry (Å, °)

*D*—H⋯*A*	*D*—H	H⋯*A*	*D*⋯*A*	*D*—H⋯*A*
C6—H6⋯O1^i^	0.93	2.52	3.331 (2)	145
N1—H1⋯O1^i^	0.85 (2)	2.30 (2)	3.1120 (18)	161 (2)

Symmetry code: (i) 
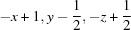
.

**Fig. 1.**
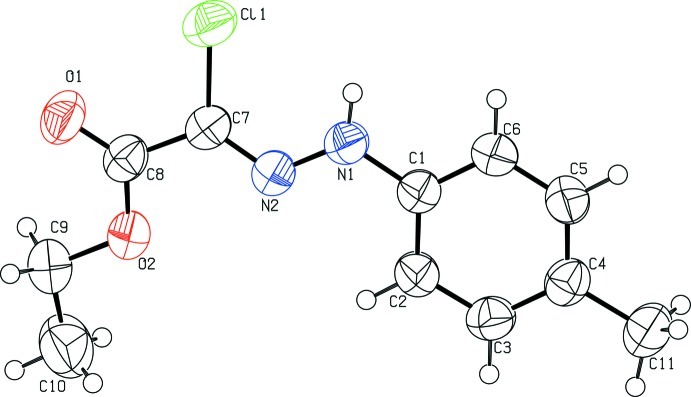
Molecular structure of the title molecule with displacement ellipsoids drawn at the 50% probability level.

**Fig. 2.**
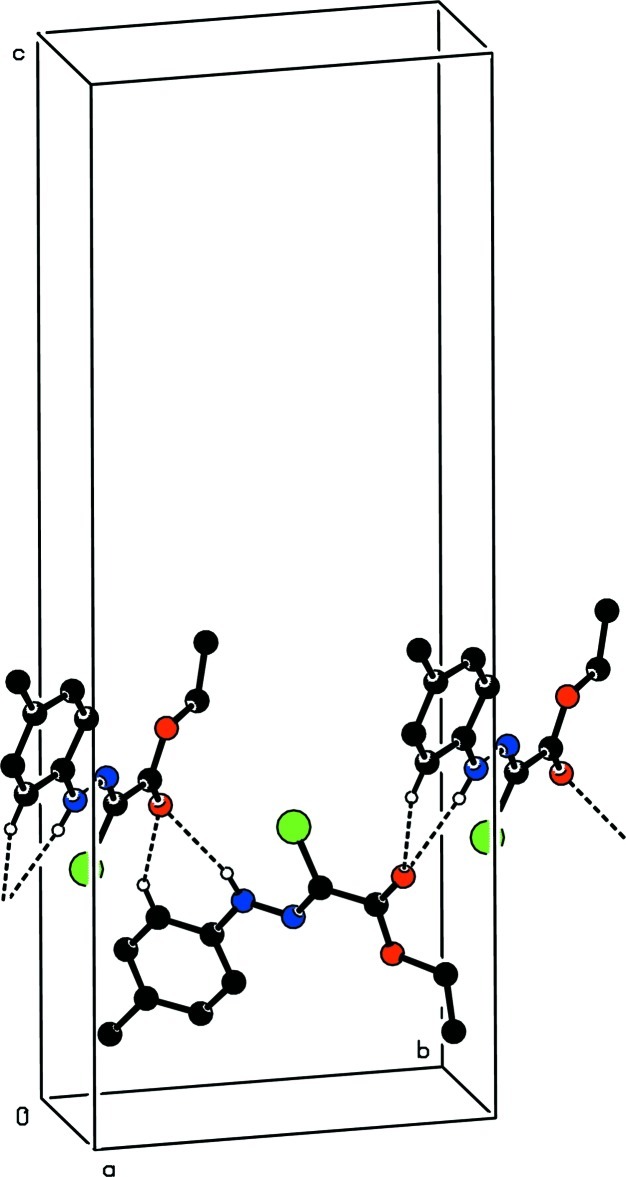
A perspective view of the crystal packing of the title compound showing N-H···O and C-H···O hydrogen bonds as dashed lines - see Table 1 for details.

## supplementary crystallographic information

### Comment

The present structure (I) is related to compounds already reported by our group, that is, (*Z*)-Ethyl 2-chloro-2-(2-(4-methoxyphenyl)hydrazono)acetate (II) (Asiri *et al.*, 2012) and 1-Chloro-1-[(4-methylphenyl)hydrazinylidene]propan-2-one (III) (Asiri *et al.*, 2011).

The title compound, Fig. 1, and II contain methyl and methoxy groups respectively at para positions of aromatic ring, which make them different from each other. The aromatic ring (C1—C6) is oriented at a dihedral angle of 9.49 (8)° with respect to the mean plane of the ester moiety (N1/N2/O1/O2 C7-Cl0; r.m.s. deviation 0.0454 Å), while the same angle in II is 3.05 (2) °. The spatial arrangements of different functional groups around the C7═N2 double bond give rise to trans isomer *i.e.**Z* conformation.

In the crystal, N—H···O and C—H···O hydrogen bonds connect the molecules along the *b* axis to form zigzag chains, enclosing six membered *R*^1^_2_(6) ring motifs - see Table. 1 and Fig. 2.

### Experimental

The molecule was synthesised according to the literature procedure (Asiri *et al.*, 2011) and recrystallized from ethanol under slow evaporation giving yellow needles.

### Refinement

The N—H H atom was located in a difference Fourier map and refined with *U*_iso_(H) = 1.2U_eq_(N). The C-bound H-atoms were included in calculated positions and treated as riding atoms: C-H = 0.93, 0.96 and 0.97 Å for CH(aromatic), CH_methyl_, and CH_methylene_ H atoms, respectively, with U_iso_(H) = k × U_eq_(parent C-atom), where k = 1.5 for CH_methyl_ H atoms and = 1.2 for other H atoms.

### Crystal data

**Table:**

C_11_H_13_ClN_2_O_2_	*F*(000) = 504
*M**_r_* = 240.68	*D*_x_ = 1.324 Mg m^−^^3^
Monoclinic, *P*2_1_/*c*	Cu *K*α radiation, λ = 1.54184 Å
Hall symbol: -P 2ybc	Cell parameters from 6552 reflections
*a* = 4.6152 (1) Å	θ = 4.4–75.9°
*b* = 9.9444 (1) Å	µ = 2.71 mm^−^^1^
*c* = 26.3152 (3) Å	*T* = 296 K
β = 90.692 (1)°	Needle, yellow
*V* = 1207.66 (3) Å^3^	0.41 × 0.14 × 0.13 mm
*Z* = 4	

### Data collection

**Table:**

Agilent SuperNova (Dual, Cu at zero, Atlas) CCD diffractometer	2436 independent reflections
Radiation source: SuperNova (Cu) X-ray Source	2224 reflections with *I* > 2σ(*I*)
Mirror monochromator	*R*_int_ = 0.019
ω scans	θ_max_ = 76.1°, θ_min_ = 4.8°
Absorption correction: multi-scan (*CrysAlis PRO*; Agilent, 2012)	*h* = −4→5
*T*_min_ = 0.692, *T*_max_ = 1.000	*k* = −12→12
9526 measured reflections	*l* = −32→33

### Refinement

**Table:**

Refinement on *F*^2^	Primary atom site location: structure-invariant direct methods
Least-squares matrix: full	Secondary atom site location: difference Fourier map
*R*[*F*^2^ > 2σ(*F*^2^)] = 0.040	Hydrogen site location: inferred from neighbouring sites
*wR*(*F*^2^) = 0.113	H atoms treated by a mixture of independent and constrained refinement
*S* = 1.06	*w* = 1/[σ^2^(*F*_o_^2^) + (0.0563*P*)^2^ + 0.2618*P*] where *P* = (*F*_o_^2^ + 2*F*_c_^2^)/3
2436 reflections	(Δ/σ)_max_ < 0.001
150 parameters	Δρ_max_ = 0.28 e Å^−^^3^
0 restraints	Δρ_min_ = −0.23 e Å^−^^3^

### Special details

**Table:**

Geometry. All esds (except the esd in the dihedral angle between two l.s. planes) are estimated using the full covariance matrix. The cell esds are taken into account individually in the estimation of esds in distances, angles and torsion angles; correlations between esds in cell parameters are only used when they are defined by crystal symmetry. An approximate (isotropic) treatment of cell esds is used for estimating esds involving l.s. planes.
Refinement. Refinement of F^2^ against ALL reflections. The weighted R-factor wR and goodness of fit S are based on F^2^, conventional R-factors R are based on F, with F set to zero for negative F^2^. The threshold expression of F^2^ > 2sigma(F^2^) is used only for calculating R-factors(gt) etc. and is not relevant to the choice of reflections for refinement. R-factors based on F^2^ are statistically about twice as large as those based on F, and R- factors based on ALL data will be even larger.

### Fractional atomic coordinates and isotropic or equivalent isotropic displacement parameters (Å^2^)

**Table:**

	*x*	*y*	*z*	*U*_iso_*/*U*_eq_	
Cl1	0.43464 (11)	0.05667 (5)	0.234472 (17)	0.07524 (19)	
O1	0.1041 (3)	0.28559 (14)	0.27188 (5)	0.0730 (4)	
O2	0.2685 (3)	0.27966 (12)	0.35220 (4)	0.0641 (3)	
N1	0.8092 (3)	−0.02052 (14)	0.31951 (5)	0.0541 (3)	
N2	0.6346 (3)	0.08135 (12)	0.32924 (5)	0.0508 (3)	
C1	0.9873 (3)	−0.07483 (15)	0.35803 (6)	0.0491 (3)	
C2	1.0086 (4)	−0.01791 (17)	0.40583 (6)	0.0579 (4)	
H2	0.9035	0.0590	0.4136	0.070*	
C3	1.1886 (4)	−0.07686 (18)	0.44203 (6)	0.0621 (4)	
H3	1.2034	−0.0379	0.4741	0.074*	
C4	1.3468 (3)	−0.19164 (17)	0.43198 (6)	0.0574 (4)	
C5	1.3267 (4)	−0.24428 (17)	0.38355 (7)	0.0626 (4)	
H5	1.4356	−0.3199	0.3755	0.075*	
C6	1.1496 (4)	−0.18777 (17)	0.34674 (6)	0.0592 (4)	
H6	1.1393	−0.2255	0.3144	0.071*	
C7	0.4608 (3)	0.12591 (16)	0.29498 (6)	0.0518 (3)	
C8	0.2603 (3)	0.23844 (16)	0.30433 (6)	0.0546 (4)	
C9	0.0576 (5)	0.3840 (2)	0.36460 (8)	0.0774 (5)	
H9A	0.1042	0.4668	0.3470	0.093*	
H9B	−0.1354	0.3560	0.3541	0.093*	
C10	0.0685 (7)	0.4052 (3)	0.41932 (10)	0.1069 (9)	
H10A	0.0065	0.3250	0.4363	0.160*	
H10B	−0.0572	0.4783	0.4281	0.160*	
H10C	0.2634	0.4263	0.4297	0.160*	
C11	1.5322 (5)	−0.2579 (2)	0.47260 (8)	0.0768 (5)	
H11A	1.4576	−0.3460	0.4797	0.115*	
H11B	1.5289	−0.2045	0.5030	0.115*	
H11C	1.7278	−0.2653	0.4609	0.115*	
H1	0.799 (5)	−0.063 (2)	0.2915 (10)	0.092*	

### Atomic displacement parameters (Å^2^)

**Table:**

	*U*^11^	*U*^22^	*U*^33^	*U*^12^	*U*^13^	*U*^23^
Cl1	0.0816 (3)	0.0890 (3)	0.0549 (3)	0.0032 (2)	−0.0103 (2)	−0.0051 (2)
O1	0.0795 (8)	0.0712 (8)	0.0680 (7)	0.0077 (6)	−0.0172 (6)	0.0174 (6)
O2	0.0687 (7)	0.0615 (7)	0.0618 (7)	0.0123 (5)	−0.0083 (5)	0.0025 (5)
N1	0.0542 (7)	0.0577 (7)	0.0502 (7)	0.0023 (6)	−0.0021 (6)	−0.0015 (6)
N2	0.0478 (7)	0.0507 (7)	0.0539 (7)	−0.0047 (5)	−0.0002 (5)	0.0055 (5)
C1	0.0459 (7)	0.0495 (7)	0.0520 (8)	−0.0049 (6)	0.0007 (6)	0.0022 (6)
C2	0.0621 (9)	0.0567 (8)	0.0549 (8)	0.0072 (7)	0.0012 (7)	−0.0032 (7)
C3	0.0664 (10)	0.0685 (10)	0.0513 (9)	0.0011 (8)	−0.0026 (7)	−0.0048 (7)
C4	0.0508 (8)	0.0597 (9)	0.0615 (9)	−0.0048 (7)	−0.0038 (7)	0.0064 (7)
C5	0.0611 (10)	0.0557 (9)	0.0709 (10)	0.0073 (7)	−0.0042 (8)	−0.0039 (7)
C6	0.0639 (10)	0.0575 (9)	0.0561 (9)	0.0035 (7)	−0.0023 (7)	−0.0083 (7)
C7	0.0502 (8)	0.0552 (8)	0.0499 (8)	−0.0079 (6)	−0.0021 (6)	0.0064 (6)
C8	0.0549 (8)	0.0529 (8)	0.0557 (8)	−0.0078 (6)	−0.0039 (7)	0.0122 (6)
C9	0.0837 (13)	0.0655 (11)	0.0826 (13)	0.0187 (10)	−0.0084 (10)	0.0009 (9)
C10	0.137 (2)	0.0947 (17)	0.0889 (17)	0.0374 (16)	0.0124 (15)	−0.0014 (13)
C11	0.0738 (12)	0.0788 (12)	0.0773 (12)	0.0022 (9)	−0.0163 (10)	0.0121 (10)

### Geometric parameters (Å, º)

**Table:**

Cl1—C7	1.7377 (16)	C4—C11	1.512 (2)
O1—C8	1.2056 (19)	C5—C6	1.380 (2)
O2—C8	1.325 (2)	C5—H5	0.9300
O2—C9	1.462 (2)	C6—H6	0.9300
N1—N2	1.3215 (19)	C7—C8	1.475 (2)
N1—C1	1.405 (2)	C9—C10	1.456 (3)
N1—H1	0.85 (2)	C9—H9A	0.9700
N2—C7	1.2788 (19)	C9—H9B	0.9700
C1—C2	1.382 (2)	C10—H10A	0.9600
C1—C6	1.384 (2)	C10—H10B	0.9600
C2—C3	1.386 (2)	C10—H10C	0.9600
C2—H2	0.9300	C11—H11A	0.9600
C3—C4	1.382 (2)	C11—H11B	0.9600
C3—H3	0.9300	C11—H11C	0.9600
C4—C5	1.380 (2)		
			
C8—O2—C9	114.85 (13)	N2—C7—Cl1	123.01 (13)
N2—N1—C1	120.49 (13)	C8—C7—Cl1	114.63 (11)
N2—N1—H1	121.5 (16)	O1—C8—O2	124.26 (16)
C1—N1—H1	117.4 (16)	O1—C8—C7	123.24 (16)
C7—N2—N1	120.52 (14)	O2—C8—C7	112.49 (13)
C2—C1—C6	119.67 (15)	C10—C9—O2	107.95 (17)
C2—C1—N1	122.27 (14)	C10—C9—H9A	110.1
C6—C1—N1	118.05 (14)	O2—C9—H9A	110.1
C1—C2—C3	119.20 (15)	C10—C9—H9B	110.1
C1—C2—H2	120.4	O2—C9—H9B	110.1
C3—C2—H2	120.4	H9A—C9—H9B	108.4
C4—C3—C2	122.08 (16)	C9—C10—H10A	109.5
C4—C3—H3	119.0	C9—C10—H10B	109.5
C2—C3—H3	119.0	H10A—C10—H10B	109.5
C5—C4—C3	117.41 (15)	C9—C10—H10C	109.5
C5—C4—C11	121.23 (17)	H10A—C10—H10C	109.5
C3—C4—C11	121.35 (17)	H10B—C10—H10C	109.5
C6—C5—C4	121.77 (16)	C4—C11—H11A	109.5
C6—C5—H5	119.1	C4—C11—H11B	109.5
C4—C5—H5	119.1	H11A—C11—H11B	109.5
C5—C6—C1	119.83 (15)	C4—C11—H11C	109.5
C5—C6—H6	120.1	H11A—C11—H11C	109.5
C1—C6—H6	120.1	H11B—C11—H11C	109.5
N2—C7—C8	122.34 (14)		
			
C1—N1—N2—C7	−176.05 (13)	C2—C1—C6—C5	1.3 (2)
N2—N1—C1—C2	−6.0 (2)	N1—C1—C6—C5	−179.88 (15)
N2—N1—C1—C6	175.21 (14)	N1—N2—C7—C8	179.40 (13)
C6—C1—C2—C3	−1.3 (2)	N1—N2—C7—Cl1	1.1 (2)
N1—C1—C2—C3	179.99 (15)	C9—O2—C8—O1	3.6 (2)
C1—C2—C3—C4	−0.4 (3)	C9—O2—C8—C7	−175.40 (14)
C2—C3—C4—C5	2.0 (3)	N2—C7—C8—O1	176.63 (15)
C2—C3—C4—C11	−177.38 (17)	Cl1—C7—C8—O1	−4.9 (2)
C3—C4—C5—C6	−1.9 (3)	N2—C7—C8—O2	−4.4 (2)
C11—C4—C5—C6	177.44 (17)	Cl1—C7—C8—O2	174.06 (11)
C4—C5—C6—C1	0.3 (3)	C8—O2—C9—C10	172.7 (2)

### Hydrogen-bond geometry (Å, º)

**Table:**

*D*—H···*A*	*D*—H	H···*A*	*D*···*A*	*D*—H···*A*
C6—H6···O1^i^	0.93	2.52	3.331 (2)	145
N1—H1···O1^i^	0.85 (2)	2.30 (2)	3.1120 (18)	161 (2)

Symmetry code: (i) −*x*+1, *y*−1/2, −*z*+1/2.
